# Mathematical modelling of thickness and temperature dependent physical aging to O_2_/N_2_ gas separation in polymeric membranes

**DOI:** 10.1039/c8ra05323e

**Published:** 2018-08-28

**Authors:** S. S. M. Lock, K. K. Lau, A. M. Shariff, Y. F. Yeong, Faizan Ahmad

**Affiliations:** CO_2_ Research Center (CO2RES), Department of Chemical Engineering, Universiti Teknologi PETRONAS Seri Iskandar Malaysia laukokkeong@utp.edu.my; School of Science, Engineering and Design, Teesside University Middlesbrough UK

## Abstract

Polymeric membranes are glassy materials at non-equilibrium state and inherently undergo a spontaneous evolution towards equilibrium known as physical aging. Volume relaxation characteristic during the course of aging is governed by the surrounding temperature in which the polymeric material is aged. Although there are studies to understand how polymeric materials evolve over time towards equilibrium at different operating temperatures, the theories have been developed merely in response to experimental observations and phenomenological theory at bulk glassy state without the implementation of sample size effects. Limited work has been done to characterize the physical aging process to thin polymeric films using reasonable physical parameters and mathematical models with incorporation of thermodynamics and film thickness consideration. The current work applies the Tait equation of states and thickness dependent glass transition temperature, integrated within a simple linear correlation, to model the temperature and thickness dependent physical aging. The mathematical model has been validated with experimental aging data, whereby a small deviation is observed that has been explained by intuitive reasoning pertaining to the thermodynamic parameters. The mathematical model has been further employed to study the gas transport properties of O_2_ and N_2_, which is anticipated to be applied in oxygen enriched combustion for generation of cleaner and higher efficiency fuel in future work.

## Abstract 2

A novel mathematical model to quantify physical aging in polymeric films with simultaneous incorporation of thermodynamics and sample size effect.

## Graphical abstract

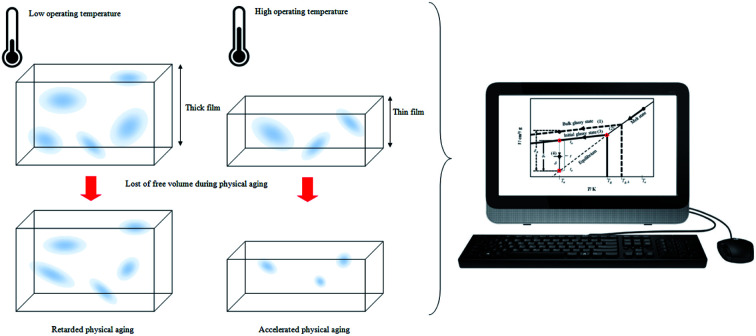

## 1. Introduction

One of the major applications employing membrane process is O_2_/N_2_ gas separation that is aimed at oxygen enrichment.^1–3^ Glassy polymeric membranes dominate membrane separation technology in an industrial scale since they have huge reproducibility for large scale production and low fabrication cost as compared to inorganic membranes,^4^ while exhibiting high gas selectivities alongside good mechanical characteristics in comparison to rubbery membranes.^5^ The commonly adapted materials for oxygen-enrichment membrane processes are polysulfone (PSF) and polyphenylene oxide (PPO) polymeric membranes.^6–9^ PSF polymeric membranes easily form thin films on membrane support surfaces, while demonstrating behaviors such as chemical inertness, good mechanical strength and stable thermal properties.^10^ On the other hand, PPO is a high performance polymeric membrane with high permeability characteristic, good mechanical properties and thermo-oxidative stability because of a low barrier to rotation and resonance stabilization of the aromatic ether bond.^11^

The disadvantage of polymeric membrane process that reduces its competitive edge is the decline permeation of gas components over time, which is commonly regarded as physical aging.^12^ This is because membranes adapted for gas separation are typically made from polymeric materials due to their superior permeability and selectivity characteristics.^13^ The polymeric membranes are amorphous and glassy materials, which are at non equilibrium condition and thereby experience a slow progression towards equilibrium naturally.^12^ The evolution towards equilibrium causes volume relaxation within the glassy polymer, further contributing to reduction of free volume that confines the permeability of gas components through the polymeric matrix.^14,15^ The aging phenomena constitutes to additional membrane area requirement to treat a given process stream than originally needed based upon design of initial membrane performance. Therefore, it is essential to elucidate the mechanism of physical aging on the performance of gas separation membranes to ensure satisfactory performance over long service periods.^16,17^ This can be achieved by designing the system to exhibit greater membrane area than required or having membrane existing in parallel to cater for a wider range of feed flow rates in order to meet sufficient oxygen recovery when aging occurs.^12^ In addition, the membrane system can be designed at membrane specification and operated at operating conditions with minimal deteriorating effect of physical aging once the process is elucidated and quantified.

The most successful mechanism to explain the volume relaxation process in thin polymeric membrane films of varying thicknesses during physical aging is the dual mode mechanism, whereby the lost of free volume has been proposed to be attributed to two phenomena, such as (1) “Lattice contraction” that describes the uniform collapse of free volume throughout the unrelaxed polymer matrix and (2) diffusion of free volume from the interior to the surface of the glassy polymer.^18–20^ Nonetheless, experimental works by Huang & Paul (2005, 2006)^21,22^ and Alsari *et al.* (2007)^23^ further reported varying behavior of physical aging in thin polymeric membranes at different external conditions, such as operating temperature and pressure. Up to date, the effect of operating conditions has not been incorporated to characterize physical aging of thin polymeric membrane films. The question of operating temperature dependent physical aging within polymeric membrane remains open and one that remains unraveled to date.

Research works devoted to mathematical modeling of thermodynamic properties of polymeric materials have been available, which plays an important role in predicting polymer behavior at various operating conditions.^24^ There are three types of thermodynamic model commonly adapted to describe the pressure–volume–temperature (PVT) characteristic of polymeric systems, which are (1) lattice model equation of states (EOS), whereby partition function of the polymer system can be obtained by counting the number of possible configurations when these segments are arranged in hypothetical cells that resemble the crystal lattice^25^ (*e.g.* Sanchez and Lacombe (SL)^26^ and non-equilibrium lattice fluid model (NELF) of Doghieri and Sarti^27^) (2) perturbation theory, whence a simple system is initially used as reference, while condition of the actual system is computed based on deviation from the reference case through introduction of certain correction or perturbation terms (*e.g.* Statistical Associated Fluid Theory (SAFT) model based upon Wertheim's ideology^28^ and Perturbed Chain (PC-SAFT))^29^ as well as (3) empirical models of polymer systems established *via* fitting with a pool of well-established experimental data (*e.g.* Tait equation^30^). In this context, the empirical model methodology has earned its wide application attributed to the simplicity and limited number of fitted parameters.^31^ Nonetheless, majority of the works have been confined to quantification of the PVT behaviour to bulk polymeric systems at a fixed time since aging in bulk system is not apparently noticeable. Probably the most successful attempt to date that has employed thermodynamics model to describe physical aging phenomena is the NELF model, which has evolved from the lattice model, whereby it has been employed extensively to model and to predict solubility of gas penetrants in glassy polymer films once the pseudo equilibrium volumetric data during the course of aging are available.^27^ Nonetheless, the NELF model has not been able to incorporate the thickness dependent physical aging that is exceptionally perceptible in thin polymeric films. The restraint constitutes to divergent from prediction of polymeric membranes employed in industrial use, which are virtually fabricated in the order of less than 1000 nm to be commercially viable in order to cater for application of large feed flux and high impurities content.^32,33^

From the review of published literatures, it is highlighted that there remains a research gap that articulates the relevancy between thermodynamic mathematical model in bulk polymeric system, which neglects the effect of sample sizes, and thickness dependent characteristic within thin polymeric membrane films. Coupling between membrane thickness and operating temperature is of utmost importance, which is essentially required in application of membrane gas separation in oxygen enriched combustion with thin selective dense polymeric material considering the adverse effect of operating temperatures.^34^ In our previous research work, we have constructed ultrathin (<100 nm) polysulfone films of varying thicknesses at its actual dimension through employment of molecular simulation tool to elucidate the effect of thickness upon confinement towards their dynamics, relaxation, physical and gas transport properties.^35,36^ Subsequently, the effect of physical aging towards separation performance and techno economic feasibility of an oxygen enriched combustion plant using thin polysulfone films has been quantified *via* variable gas transport behaviour modelled through the dual mode mechanism model.^20^ Nonetheless, all the works have been confined to assumption of ambient operating temperature of 308.15 K (35 °C) while the question of temperature and thickness dependent physical aging continues to be unresolved.

The novelty of current work is to use thermodynamic approach to describe temperature and thickness dependent physical aging simultaneously through mathematical modelling. To the best of our knowledge, this is the first pioneering work that incorporates both temperature and thickness dependent effect of physical aging to fulfill previous research work that has been devoted to either modeling of thickness dependent physical aging in thin film through lost of free volume *via* dual mode mechanism or modelling of temperature dependent physical aging with availability of pseudo equilibrium volumetric data for polymeric film at a fixed dimension *via* the NELF model. In this work, a simple linear correlation has been employed to tackle aging behavior at different aging temperatures and film thicknesses to provide physical interpretation towards the aging phenomena from a thermodynamic point of view that stems from the thickness dependent glass transition temperature and relaxation time to achieve equilibrium. The current work applies the Tait equation of states and thickness dependent glass transition temperature, integrated within a simple linear correlation, to achieve the simultaneous effect of modelling the temperature and thickness dependent physical aging. The well-known Tait equation has been selected as thermodynamic model in present study since it has been successfully utilized to characterize PVT properties of bulk polymer at the glass and melt conditions accurately over a wide range of operating conditions.^37^ The well-established Tait equation has earned wide acknowledgement due to its simplicity, which enables parameters of the empirical model to be found conveniently in varying published literatures to be employed in physical aging model of present work.^37^ On the other hand, the thickness dependent glass transition temperature has been employed since it is one of the most notable characteristic that exhibits deviation of actual properties of the thin film at dimension upon confinement in comparison to its bulk counterpart. To demonstrate accuracy of the proposed mathematical model, it has been validated using published experimental aging data at different operating temperatures and film thicknesses for PSF and PPO to reveal applicability of the methodology for different classes of glassy polymers. The mathematical model is found to provide good approximation and reasonable intuitive explanation pertaining to the thermodynamic evolution of polymeric materials with varying thicknesses and operating temperatures during physical aging. It is worth noting that the mathematical model is merely limited to non condensable gas pairs (*e.g.* O_2_/N_2_) since separation of gases that deals with condensable gas (*e.g.* CO_2_/CH_4_) has been reported to interact with functional group of polymeric chain, which consequently swells the membrane matrix and affects the free volume alteration during physical aging.^38,39^ Hence, in present work, the gas transport properties of O_2_ and N_2_ over the course of aging at varying operating temperatures are elucidated, which is hopeful to be applied in oxygen enriched air through polymeric membranes for production of higher efficiency combustion.

## 2. Methodology

**Fig. 1:**
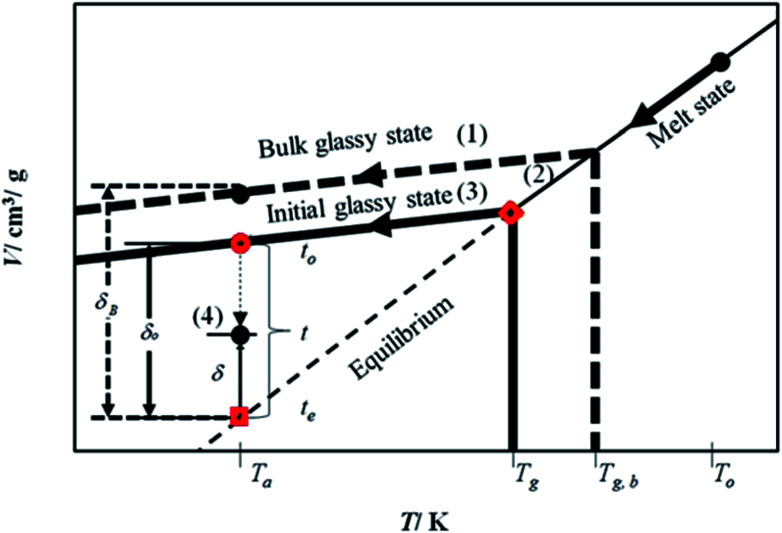
Schematic representation of physical aging process based on specific volume, *V*, and departure from equilibrium, *δ*, within glassy material at aging temperature, *T*_a_, below the glass transition temperature, *T*_g_, and bulk glass transition temperature, *T*_g,b_, for development of the linear correlation as a function of time, *t*.

The mathematical model is developed based on the solution algorithm as presented in Fig. 1.

From Fig. 1, it is depicted that the methodology is comprised of five following steps.

(1) Slope of thermodynamic line characterizing the bulk glass state.

(2) Thickness dependent glass transition temperature.

(3) Equation of thermodynamic line characterizing the thin glassy state.

(4) Linear correlation of physical aging based on deviation from equilibrium.

The description of each step is provided in the next subsections.

### 2.1 Slope of the thermodynamic line characterizing the bulk glassy state

In this work, the Tait equation, which has been demonstrated to be particularly successful to provide a simple and convenient mathematical characterization of PVT behaviour for a large number of rubbery and glassy polymers, has been adopted to describe the compressibility characteristic of the bulk polymeric matrix.^40^ In addition, it has been demonstrated in published literatures by Huang *et al.* (2006),^21^ whereby their experimental aging data would be adapted to validate the developed methodology, that the Tait equation is of sufficient accuracy to characterize the specific volume of their fabricated polymers. The Tait equation has generally adopted the common form, such as that presented in eqn (1).^30^1
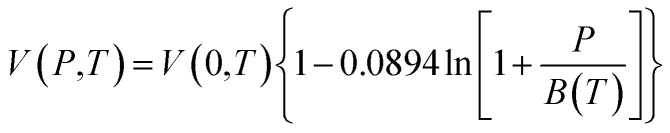
Whereby *V*(*P*,*T*) represents the specific volume of the polymer at a particular temperature, *T*, and pressure, *P*, of interest, *V*(0,*T*) corresponds to the volume–temperature isotherm at zero pressure while *B*(*T*) is the Tait parameter.

In order to evaluate the physical aging of polysulfone (PSF) and polyphenylene oxide (PPO) under different operating temperatures, the correlations for volume–temperature isotherm at zero pressure, *V*(0,*T*), and Tait parameter, *B*(*T*), have been employed from Zoller's work.^41–43^ In his research, the pressure–volume–temperature (PVT) properties of bulk PSF and PPO are studied experimentally in a wide range of pressures and temperatures to derive mathematical correlations based on the well-known Tait equation, such as that provided in eqn (2)–(5) for the initial glass and eqn (6)–(9) for the melt condition.

Initial glass:

PSF:

2 *V*(0,*T*) = 0.8051 + 1.756 × 10^−4^*T*

3 *B*(*T*) = 4408 exp(−1.543 × 10^−3^*T*)

PPO:

4 *V*(0,*T*) = 0.9348 exp(2.09 × 10^−4^*T*)

5 *B*(*T*) = 3379 exp(−2.00 × 10^−3^*T*)

Melt:

PSF:

6 *V*(0,*T*) = 0.7644 + 3.419 × 10^−4^*T* + 3.126 × 10^−7^*T*^2^

7 *B*(*T*) = 3731 exp(−3.757 × 10^−3^*T*)

PPO:

8 *V*(0,*T*) = exp[−2.475 × 10^−1^ + 2.151 × 10^−5^(*T* + 273.15)^1.5^]

9 *B*(*T*) = 2323 exp(−4.29 × 10^−3^*T*)

In eqn (2)–(9), temperature, *T*, is in °C.

Nonetheless, since the initial specific volume of the bulk state, *V*_initial_glass,b_(*p*,*T*_a_), at operating pressure *p* and aging temperature *T*_a_, is dependent upon formation history of the sample, the applicability of the Zoller's expression^41–43^ to validate the PSF and PPO membrane prepared in Huang & Paul (2004) work has been further discussed and evaluated.^44^ In Zoller's work (1978, 1982), the measurement of specific volume at different operating temperature and pressure has been conducted directly after annealing at high temperature under a dry nitrogen atmosphere.^41,43^ The anneal treatment is aimed to remove residual stresses captured within the polymer while minimizing interference from the surroundings through exposure to an inert gas throughout. This characterizes the fresh sample before loading into the PVT apparatus for determination of the empirical model. Any effects of physical aging during the measurement procedure have been assumed to be negligible since it has been evidenced in previous published literature that the phenomena is retarded in thick samples over a short time scale.^45^ As for the PSF polymeric film adopted to validate the developed mathematical model, Huang & Paul (2004) have utilized the thermoreversibility characteristic of aging and erasure of any prior thermal or pressure histories of the samples by reheating above the glass transition temperature and annealing with a N_2_ purge before quenching to the aging temperature of interest.^44^ The procedure has been supported by several other published literature to be eligible in order to remove any accumulated histories attained in glassy materials, *i.e.*, previous thermal history, orientation and other effects.^46^ Therefore, the polymeric samples have been deduced to be carefully conditioned to imprint a fresh history prior to initiating the aging test.

Furthermore, in order to ensure that the bulk PSF films in both studies are comparable to adopt the same PVT characteristic, several properties of the PSF films, such as density, *ρ*, and glass transition temperature, *T*_g_, are summarized in Table 1.

**Table 1:** Comparison of density, *ρ*, and glass transition temperature, *T*_g_, properties of bulk PSF sample

Reference	*ρ*/g cm^−3^		*T*_g_/°C	
	PSF	PPO	PSF	PPO
Zoller	1.234^a^	1.064^b^	185–186 (ref. 41)	205 (ref. 43)
Huang & Paul	1.240^c^	1.067^c^	186 (ref. 44)	210 (ref. 44)

a Experimental value by Zoller (1978) at 27 °C (ref. 41).

b Experimental value by Zoller (1982) at 25 °C (ref. 43).

c Experimental value by Huang & Paul (2004) at 25 °C (ref. 44).

It is depicted from Table 1 that properties of the PSF and PPO bulk films from both studies are similar, which supports that the histories of the samples have been preset to fresh conditions. The experiments have been conducted with care to remove the interference of prior histories on the polymeric samples and therefore the empirical models can be sufficiently adopted in current study.

By adopting Zoller's expressions to determine the specific volumes of the initial glass and melt at the corresponding temperature of interest, the slope of the thermodynamic line characterizing the bulk polymer (Line (1) in Fig. 1), *m*_b_, is computed based on eqn (10).10
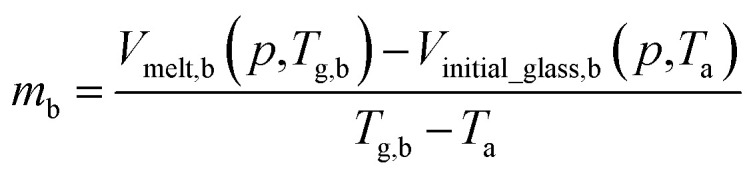
Whereby *V*_melt,b_(*p*,*T*_g,b_) is the specific volume of bulk polymer melt at operating pressure, *p*, and bulk glass transition temperature, *T*_g,b_, while *V*_initial_glass,b_(*p*,*T*_a_) is its specific volume at operating pressure, *p*, and aging temperature, *T*_a_. *V*_initial_glass,b_(*p*,*T*_a_) has been computed based on eqn (1), (2) and (3) for PSF and eqn (1), (4) and (5) for PPO respectively at their corresponding aging temperature of interest. The pressure of the system, *p*, is kept constant at 2 atm throughout the work to be consistent with experimental physical aging data, which has been conducted and collected at the particular operating pressure.^47^ Therefore, physical aging is independent of operating pressure, and thus the effect operating temperature can be isolated conveniently in current study. The value of *T*_g,b_ for bulk PSF and PPO has been experimentally determined to be 459.15 K (186 °C) and 483.15 K (210 °C) from Huang & Paul original work adopting differential scanning calorimetry (DSC).^48^

### 2.2 Thickness dependent glass transition temperature

The deviation of depressed glass transition temperature in thin films, T_g_(*l*), in comparison to glass transition temperature of bulk state, *T*_g,b_, which subsequently affects the temperature dependent specific volume, is depicted in Line (2) of Fig. 1. The actual glass transition temperature, *T*_g_(*l*), of the thin polymeric membrane under study has been calculated adopting Keddie's thickness dependent glass transition temperature, as depicted in eqn (11).^49^11
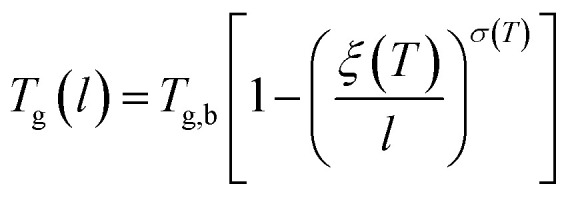
Whereby *T*_g,b_ is the bulk glass transition temperature, *l* is thickness of the polymeric film, *T*_g_(*l*) is the thickness dependent glass transition temperature of the polymer, while *ζ*(*T*) and *σ*(*T*) correspond to parameters that describe the characteristic length of the specific polymer and the exponent variable respectively, which have been both determined to be dependent upon the surrounding temperature.

### 2.3 Equation of thermodynamic line characterizing the thin glass state

In order to obtain equation of the thermodynamic line characterizing the actual glassy state in thin films (Line (3) of Fig. 1), slope of the line, *m*_actual_, is assumed to be the same as the bulk polymer, which has been supported in work by Zoller that the slope of thermodynamic line at varying operating conditions are approximately similar,^41–43^ such as that depicted in eqn (12).

12 *m*_actual_ = *m*_b_

The specific volume of the thickness dependent PSF at the actual glass transition temperature, *T*_g_(*l*), under melt condition, *V*_melt_(*p*,*T*_g_(*l*)), (shown in 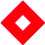 of Fig. 1) has been computed adopting eqn (1), (6) and (7). Similarly, for PPO films, *V*_melt_(*p*,*T*_g_(*l*)) has been calculated based upon eqn (1), (8) and (9). The constant of the thin line, *C*_actual_, can be conveniently calculated employing the slope of the line, *m*_actual_, and a single point that lies on it, [*T*_g_(*l*),*V*_melt_(*p*,*T*_g_(*l*))], such as that depicted in eqn (13).

13 *C*_actual_ = *V*_melt_(*p*,*T*_g_(*l*)) − *m*_actual_*T*_g_(*l*)

Therefore, equation of the actual thermodynamic line at the initial glassy state of thin film (Line (3) of Fig. 1) for temperature, *T*, below the glass transition temperature, *T*_g_(*l*), is derived in eqn (14).

14 *V*_initial_glass_(*p*,*T*) = *m*_actual_*T* + *C*_actual_

Specific volume of the initial glassy state, *V*_initial_glass_, (shown in 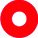 of Fig. 1) is computed by substituting the aging temperature of interest, *T*_a_, into eqn (14). This value specifies the initial specific volume of the PSF polymeric film, which gradually undergoes physical aging process, constituting to the reduction in free volume within the polymeric matrix over the course of aging.

### 2.4 Linear correlation of physical aging based on deviation from equilibrium

It has been reported in previous work by Huang & Paul (2005) that gas permeability over time can be represented by a linear log–log correlation,^22,48^ such as that depicted in (15).

15 ln *P*(*t*,*T*) = *x*(*T*)ln *t* + *y*(*T*)

In (15), *P*(*t*,*T*) is the time and temperature dependent gas permeability while *x*(*T*) and *y*(*T*) characterize the slope and constant of the intercept in the log–log plot at different operating temperature *T* respectively. In addition, the permeability at time, *t*, and temperature, *T*, *P*(*t*,*T*), can be similarly described employing an exponential correlation as a function of the fractional free volume, *f*(*t*,*T*), such as that provided in (16).^50^16
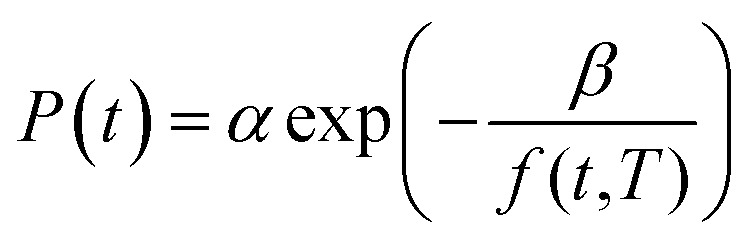


In (16), *α* and *β* are constants of the permeability correlation for gas component, which represent the pre-exponential factor and minimum volume of fluctuation needed for diffusion jump respectively. In addition, the pre-exponential factor *α* in eqn (16)^51,52^ has been reported to exhibit Arrhenius relationship with respect to operating temperature, such as that depicted in eqn (17).17
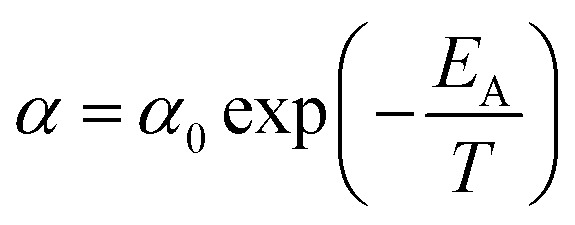


In eqn (17), *α*_0_ is pre-exponential constant while *E*_A_ is enthalpy change associated to effect of temperature to alteration in gas permeability. The Arrhenius relationship reduces the temperature dependent gas permeability to eqn (18).18
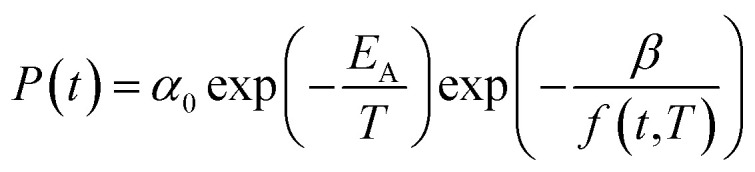


In order to quantify the amount of free space that characterizes the efficiency of chain packing, the time dependent fractional free volume, *f*(*t*,*T*), is a useful and commonly employed parameter to elucidate the morphology and configuration of polymeric membranes, whereby the definition is provided in (19).19
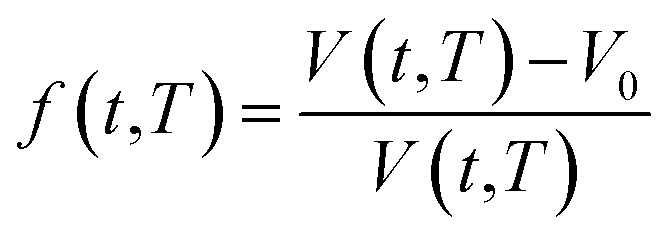


In eqn (19), *V*(*t*,*T*) is specific volume of the glassy polymeric membrane at a specific temperature and aging time, while *V*_0_ is the specific occupied volume of polymer chain, which can be determined adopting various group contribution methodologies, such as that proposed by Bondi,^53^ as depicted in (20).20
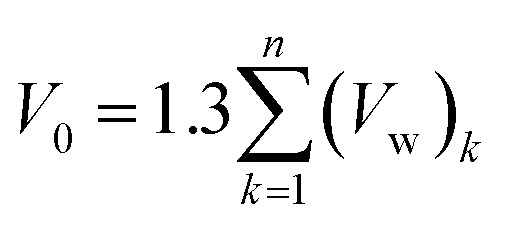
where *n* is the total number of groups into which the repeat unit structure of a polymer is divided, while *V*_w_ is the van der Waals volume of the group, such as that proposed by Van Krevelen (1990).^54^ For PSF and PPO, *V*_0_ has been determined to be 0.6903 g cm^−3^ and 0.764 g cm^−3^ to be utilized consistently throughout the work. Through substitution of (16) and (19) into (15), followed by some mathematical treatments, (21) is derived, which is resemblance of the linear behavior of a normalized specific volume of glassy polymer, 
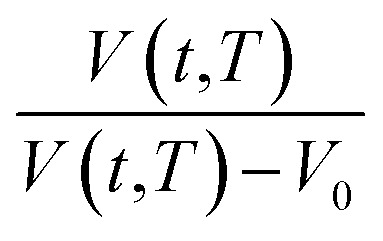
, as a function of natural logarithmic of aging time, *t* (Line (4) of Fig. 1).21
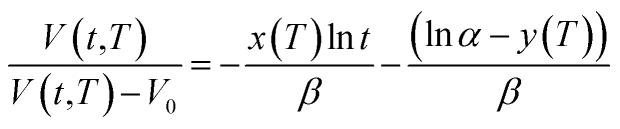


**Fig. 2:**
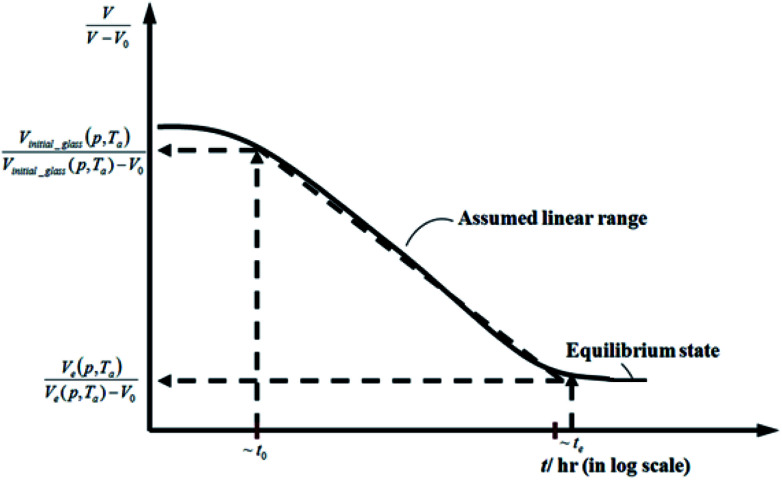
Schematic plot of linear approximation of normalized specific volume, 
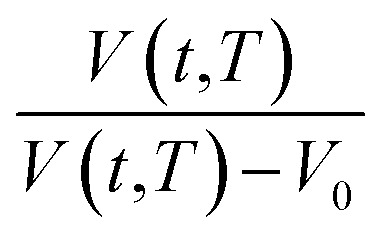
, as a function of aging time, *t*, in natural logarithmic scale.

Based on published literature by Huang & Paul (2005),^22,48^, (15) is a linear correlation with negative slope, which implies *x*(*T*) carries a negative weightage.^22,48^ The effect of 
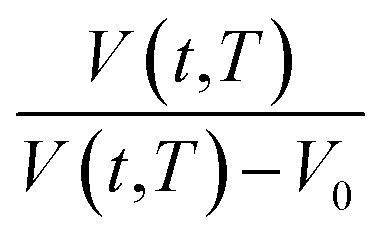
 as a linear relationship with respect to ln *t* is depicted in Fig. 2.

In this work, the linear behavior in (21) has been adopted to characterize the physical aging process under different aging temperatures, *T*_a_, by exhibiting temperature dependent specific volume at the initial glassy state, *V*_initial_glass_(*t*_0_,*T*_a_), and equilibrium *V*_e_(*t*_e_,*T*_a_), as well as time to achieve equilibrium condition, *t*_e_. *V*_e_(*t*_e_,*T*_a_) (shown in 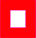 of Fig. 1) has been calculated employing eqn (1), (6) and (7) for PSF and eqn (1), (8) and (9) for PPO by substituting the aging temperature of interest, *T*_a_, into the aforementioned equations. On the other hand, the equilibrium time, *t*_e_, that characterizes the total time for the polymeric matrix to relax before achieving equilibrium specific volume is described using a modified Williams–Landel–Ferry (WLF)-shift equation. The WLF expression has been originally adapted to characterize the temperature dependence of relaxation mechanisms in amorphous polymers.^55^ In this work, relaxation has been proposed to be correlated to the time to achieve equilibration since it characterizes the rate in which glassy polymer state attains its ultimate hypothetical melt state whereby relaxation is finally retarded. The original WLF-shift equation is depicted in eqn (22), while adaptation of the modified WLF expression to determine the equilibrium time, *t*_e_, is provided in eqn (23).22
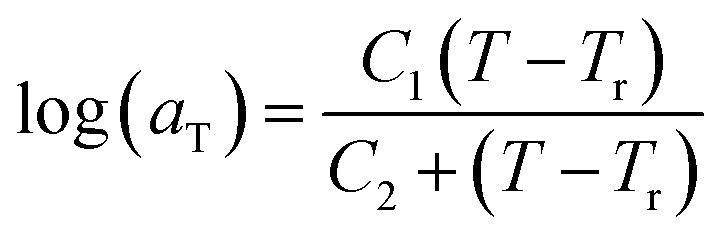
23
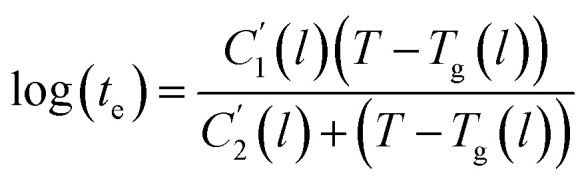


In the WLF equation as depicted through eqn (22), *a*_T_ is the shift factor that describes the relaxation mechanism of the polymer, *C*_1_ and *C*_2_ are constants respectively, while *T* and *T*_r_ correspond to the temperature and reference temperature. On the other hand, in the revised WLF expression as presented in eqn (23), *t*_e_ is the total time for the polymeric matrix to relax before achieving equilibrium specific volume, 
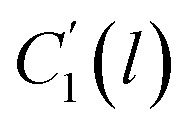
 and 
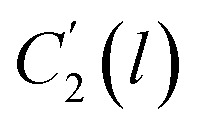
 are the thickness dependent constants, whilst *T* and *T*_g_(*l*) correspond to the temperature and glass transition temperature. The slope of the aging line, *m*_aging_, in Fig. 2 has been calculated adopting eqn (24).24
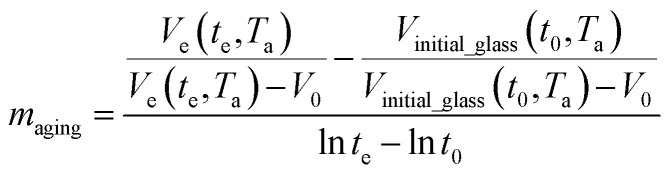
Whereby *t*_0_ is the initial aging time, which has been assumed to be 1 h constantly throughout the work, so that constant of the aging line, *C*_aging_, is simplified to be 
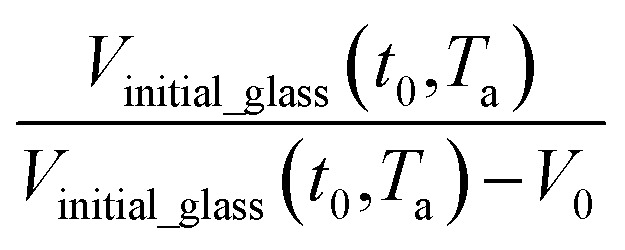
. In Huang & Paul work (2004), time zero for physical aging has been taken as the point when the thin polymeric film samples have been removed from the oven after being brought to the aging temperature.^44^ After that, the samples have to be mounted on the silicon wafer surface to obtain the average measurement value *via* ellipsometry by acquiring at least three refractive index specifications for each film sample thickness and aging temperature.^44^ Hence, instead of considering the 1 h assumption as a time interval from initiation of physical aging, it can be deduced that the assumption has been taken as the average time for preparation of samples in the sampling chamber before the preliminary measurement. Similar assumption has been reported in original work by Huang *et al.* (2006), whereby the physical aging data were only approximated at time zero since actual measurement had been conducted after a series of well carried out protocols to prepare samples on the instrument.^21^ Therefore, the linear correlation characterizing the aging process can be conveniently derived at any aging time, *t*, such as that provided in eqn (25).25
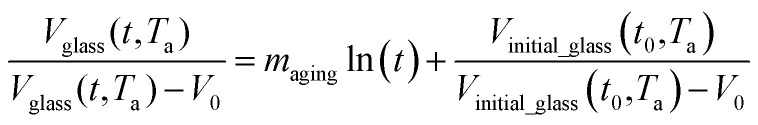


Subsequently, fractional free volume and gas permeability at varying aging time and temperature has been computed based on eqn (19) and (16) respectively.

## 3. Results and discussion

**Fig. 3:**
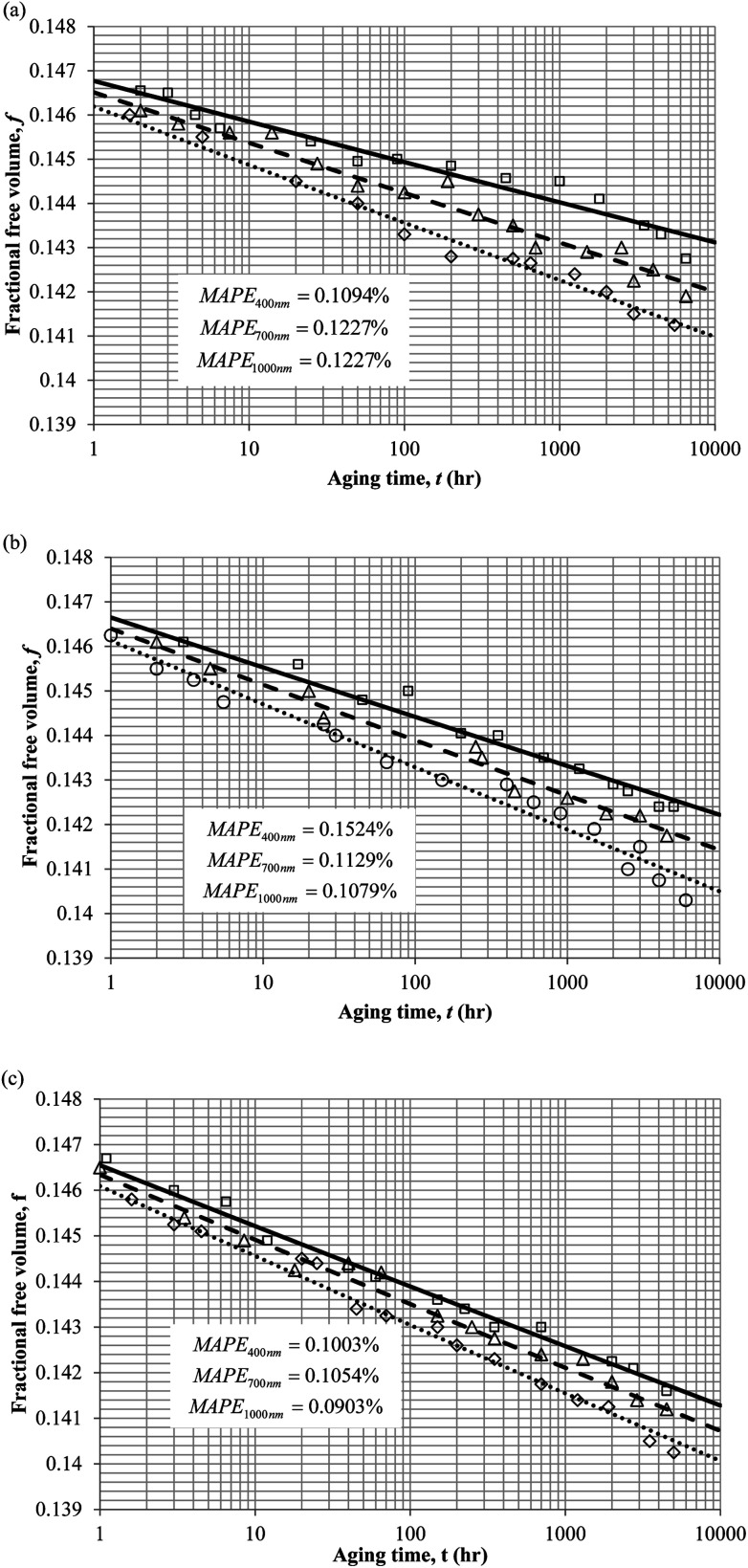
Model validation with fractional free volume, *f*, data by Huang *et al.* (2005) of Polysulfone membrane film during aging time, *t*, with thicknesses of ∼400 nm (○), ∼700 nm (△) and ∼1000 nm (□) with simulation results of ∼400 nm (⋯), ∼700 nm (- - -) and ∼1000 nm (—) at temperature (a) 35 °C (b) 45 and (c) 55 °C.

**Fig. 4:**
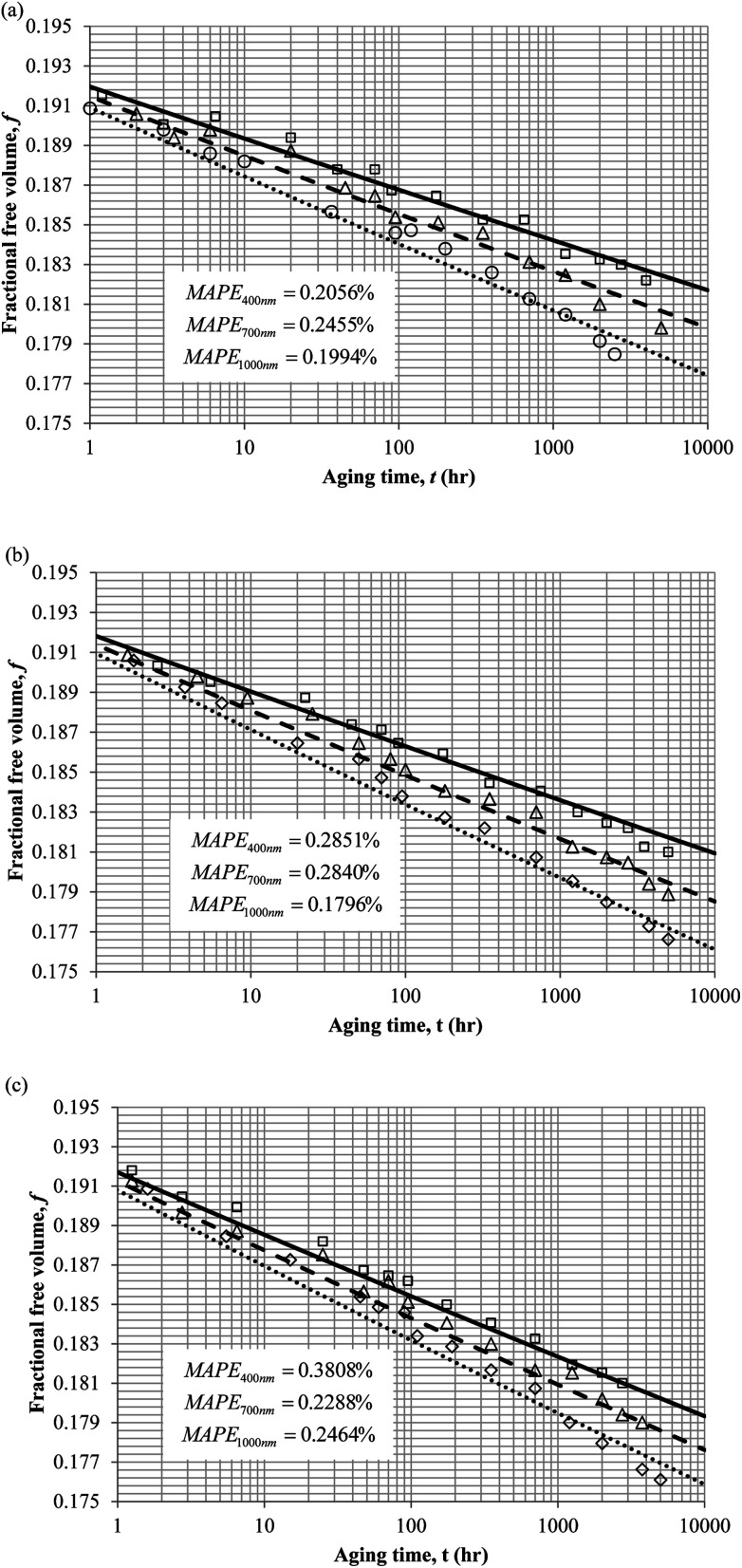
Model validation with fractional free volume, *f*, data by Huang *et al.* (2005) of polyphenylene oxide (PPO) membrane film during aging time, *t*, with thicknesses of ∼400 nm (○), ∼700 nm (△) and ∼1000 nm (□) with simulation results of ∼400 nm (⋯), ∼700 nm (- - -) and ∼1000 nm (—) at temperature (a) 35 °C (b) 45 and (c) 55 °C.

To demonstrate applicability of the developed mathematical model, it has been validated adapting physical aging experimental data of free volume for PSF and PPO membrane film with varying thicknesses (∼400 nm, ∼700 nm and ∼1000 nm) at 35 °C, 45 °C and 55 °C conducted by Huang *et al.* (2005), as depicted in Fig. 3(a), (b) and (c) and Fig. 4(a), (b) and (c) respectively.^22,48^ To quantify accuracy of the simulated free volume, the value of Mean Absolute Percentage Error (MAPE) has been provided, whereby the definition has been provided in (26), in which *x*_sim_ and *x*_exp_ correspond to simulated and experimental data respectively, while *N*_exp_ is the number of collected experimental data.26
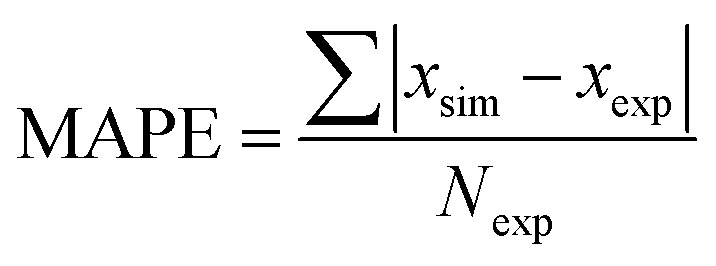


The physical parameters employed to fit the experimental observation for Keddie's *et al.* thickness dependent glass transition temperature expression and the temperature dependent revised WLF expression are provided in Tables 2 and 3 respectively. This approach successfully isolates the effect of size dependent glass transition temperature adopting temperature dependent parameters and thermodynamic relaxation behaviour employing thickness dependent variables on the latter. The parameters have been approximated *via* curve fitting by optimization in Matlab® 2013 through minimization of summation of squares of errors between the experimental data and the simulation model.

**Table 2:** Model parameters deduced by fitting aging data for thin films of PSF polymer at different aging temperature, *T*_a_, for the thickness dependent, *l*, glass transition temperature expression proposed by Keddie *et al.*^49^ (eqn (11))

*l*/nm	*ζ*(*T*) (×10^−8^) (nm)						*σ*(*T*) (dimensionless)					
	PSF			PPO			PSF			PPO		
	35 °C	45 °C	55 °C	35 °C	45 °C	55 °C	35 °C	45 °C	55 °C	35 °C	45 °C	55 °C
∼400												
∼700	42.1	2.19	0.502	2.50	0.90	0.25	0.190	0.140	0.115	0.200	0.170	0.140
∼1000												

**Table 3:** Model parameters deduced by fitting aging data for thin films of PSF polymer at different membrane thickness, *l*, for the temperature dependent revised WLF (eqn (23))

*l*/nm	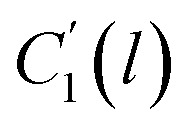 (log h)						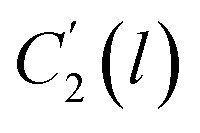 (K)					
	PSF			PPO			PSF			PPO		
	35 °C	45 °C	55 °C	35 °C	45 °C	55 °C	35 °C	45 °C	55 °C	35 °C	45 °C	55 °C
∼400	2800			3000			−1.260 × 10^4^			−9.900 × 10^3^		
∼700	3100			3125			−1.250 × 10^4^			−9.000 × 10^3^		
∼1000	3500			3255			−1.236 × 10^4^			−8.200 × 10^3^		

It is illustrated from Fig. 3 and 4 that the mathematical model is able to provide good characterization of the physical aging process at various operating temperature from a thermodynamic point of view demonstrated through the small MAPE values (<0.2% for PSF and <0.4% for PPO) between model prediction and experimental data. The small deviation can be attributed to experimental limitations, whereby the fractional free volume can only be obtained through ellipsometry technique with precision restraints of instruments. It is found from Table 2 that the value of *ζ*(*T*) is bigger at lower aging temperature, which is consistent to observation reported by Forrest & Mattsson.^56^ As highlighted earlier, the *ζ*(*T*) parameter is a quantitative representation of the characteristic length, which corresponds to a length scale of the cooperative rearrangement due to polymer segmental relaxation. The size of energy barrier grows with decreasing temperature since the polymeric matrix inherits a reduced energetic state on which the rates of all configurational rearrangements depend to achieve equilibrium. The enhanced resistance contributes to increment in length scale or a growing number of units that must be collectively be activated for motion to occur at lower operating temperature.^57,58^ Similarly, the same observation is noticeable to the exponent parameter, *σ*(*T*), whereby the variable decreases with increment in the aging temperature. The larger exponent parameter when operating temperature is reduced has been attributed to greater deviation from the bulk glass transition temperature.

**Fig. 5:**
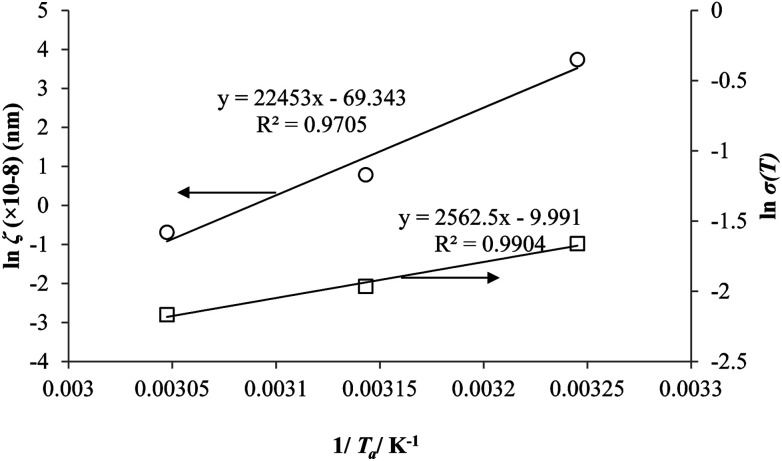
Arrhenius temperature plots for *ζ*(*T*) (○) and *σ*(*T*) (□) of free volume data for PSF.

**Fig. 6:**
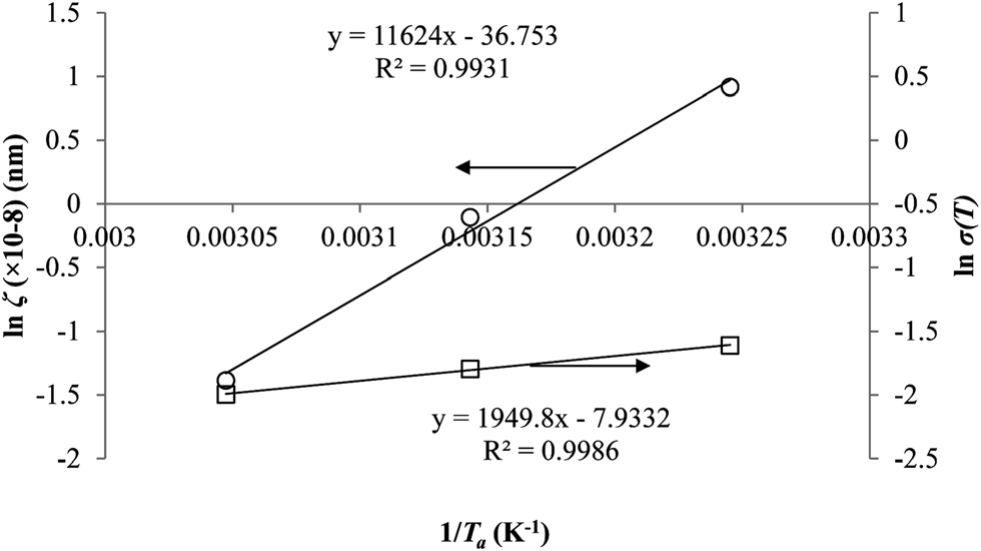
Arrhenius temperature plots for *ζ*(*T*) (○) and *σ*(*T*) (□) of free volume data for PPO.

Coincidently, when plotting the logarithmic of both parameters against the reciprocal of the aging temperature, they were found to exhibit a relatively linear correlation, which is evident that the relaxation at the thickness dependent glass transition temperature is somehow governed by an Arrhenius relationship, such as that depicted in Fig. 5 for PSF and Fig. 6 for PPO.

An example of the form of Tool–Narayanaswamy–Moynihan (TNM) model that describe the relaxation characteristic by incorporating the effect of temperature and structure is provided in eqn (27).^59–61^27
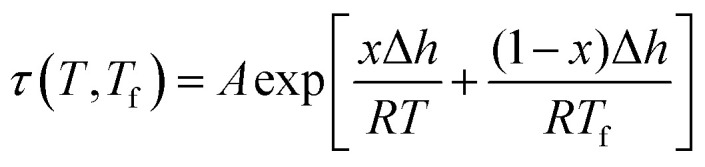
Whereby *τ* is the relaxation time, *A* is the pre-exponential factor, Δ*h* is the activation energy, *R* is the ideal gas constant, *T* and *T*_f_ is the operating and fictive temperature that characterizes the contribution of temperature and structure to the relaxation, while *x* is the partitioning parameter that determines the boundaries of the degree of non-linearity. At the glass transition temperature with equilibrium structure whereby *T*_f_ = *T*, eqn (27) is reduced to the Arrhenius form, which explains the linear correlation observed in Fig. 5 and 6. In addition, it is found that slope of the *ζ* and *σ* parameter with respect to the effect of aging temperature is bigger for PSF in Fig. 5 as compared to PPO in Fig. 6. This is because PSF is a polymer that exhibits more sensitivity to temperature in comparison to PPO.^22^

**Fig. 7:**
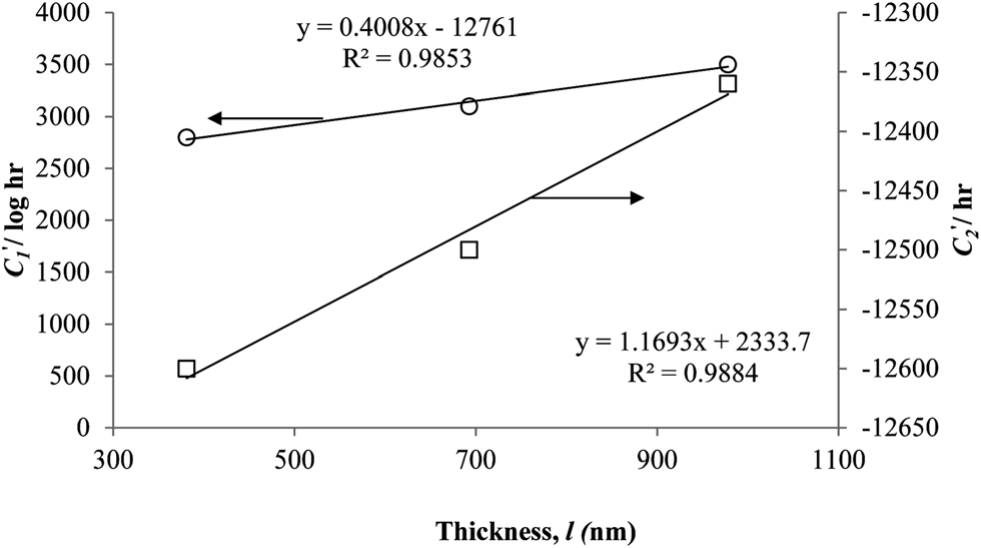
Effect of film thickness, *l*, to 
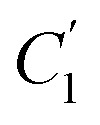
 (○) and 
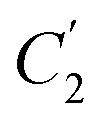
 (□) in the revised WLF equation for PSF.

**Fig. 8:**
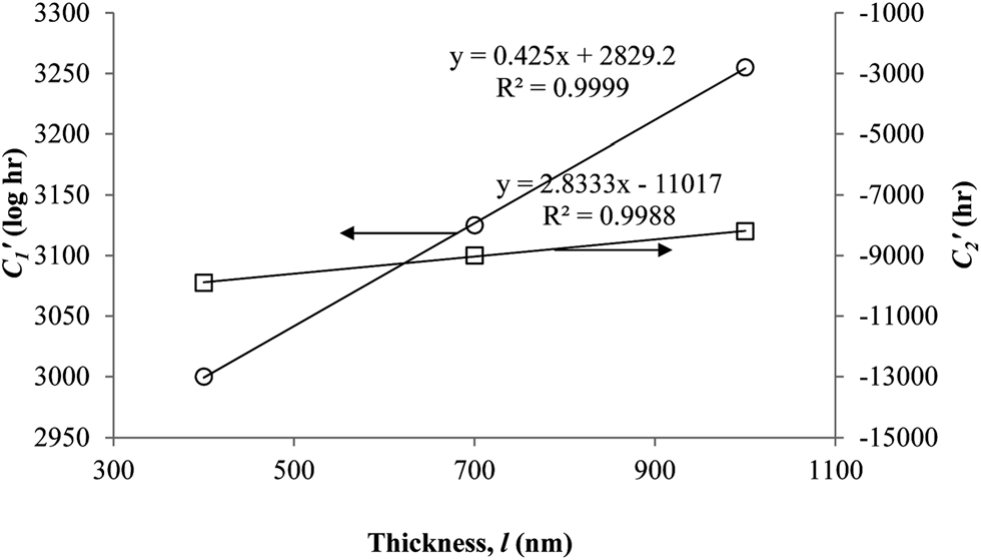
Effect of film thickness, *l*, to 
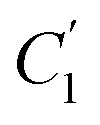
 (○) and 
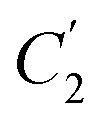
 (□) in the revised WLF equation for PPO.

In order to evaluate applicability of the parameters in the revised WLF equation, as summarized in Table 3, it has been plotted against the film thickness, as provided in Fig. 7 for PSF and Fig. 8 for PPO membrane film. It is depicted that the parameters, 
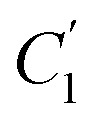
 and 
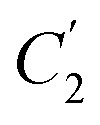
, demonstrate a relatively linear correlation with respect to the thickness of polymeric membrane film, which suggests that the methodology in present work can be conveniently used to derive empirical relationship in order to interpolate and predict physical aging performance in between the thicknesses. In addition, it has been depicted that slope of the parameters, 
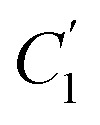
 and 
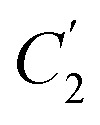
, with respect to film thickness is higher for PPO in Fig. 8 as compared to PSF in Fig. 7. The observation has been attributed to higher aging rate in PPO, which can be rationalized through its higher fractional free volume that enables a driving force of physical aging when film thickness is confined.^62^

**Fig. 9:**
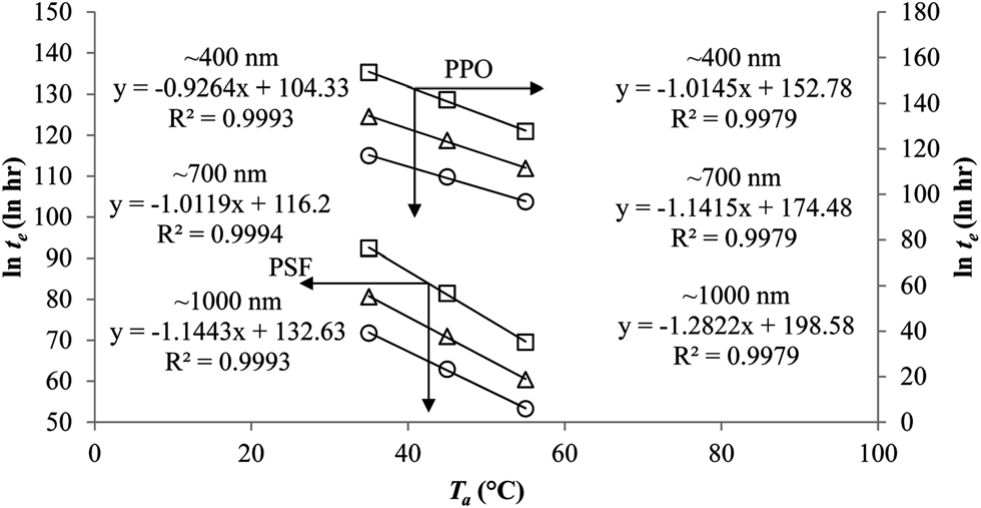
Temperature dependence of the time to reach equilibrium, *t*_e_, for PSF and PPO membrane film with varying thicknesses of ∼400 nm (○), ∼700 nm (△) and ∼1000 nm (□) at different aging temperature, *T*_a_.

In addition, the natural logarithmic of the time to achieve equilibrium, ln *t*_e_, is plotted against the aging temperature at different polymeric film thicknesses in Fig. 9. The temperature dependence of time to reach equilibrium is a linear function, which is consistent with the plot reported by Simon *et al.* (1998).^63^ At the same aging temperature, the time to achieve equilibrium, *t*_e_, is longer for thicker polymeric membrane film, which is consistent with accelerated aging observed in thinner polymers.^64^ The accelerated aging has been rationalized by higher possibility of free volume diffusion and escape from the surface in a smaller dimension, contributing to faster reestablishment of the thermodynamic equilibrium and hence shorter equilibrium time in comparison to its bulk counterpart.^18^ In addition, the equilibration time is also longer at lower aging temperature, which has been attributed to lower energetic state of polymeric chains that contributes to retarded relaxation, further constituting to prolonged time period to achieve the final equilibrium condition.

Ultimately, the effect of aging temperature induced physical aging to polymeric membranes has been investigated from the aspect of gas permeability, which has been modeled according to eqn (16), with the physical parameters been summarized in Table 4 for oxygen and nitrogen gases respectively.

**Table 4:** Values of constants in gas permeability-free volume empirical model

*T*/°C	*α* (barrer) (×10^8^)			*β*/dimensionless		
	PSF		PPO	PSF		PPO
	O_2_	N_2_	O_2_	O_2_	N_2_	O_2_
35	1.260	3.100	0.2550			
45	1.550	4.100	0.2700	2.61	2.98	2.66
55	1.675	4.800	0.2825			

**Fig. 10:**
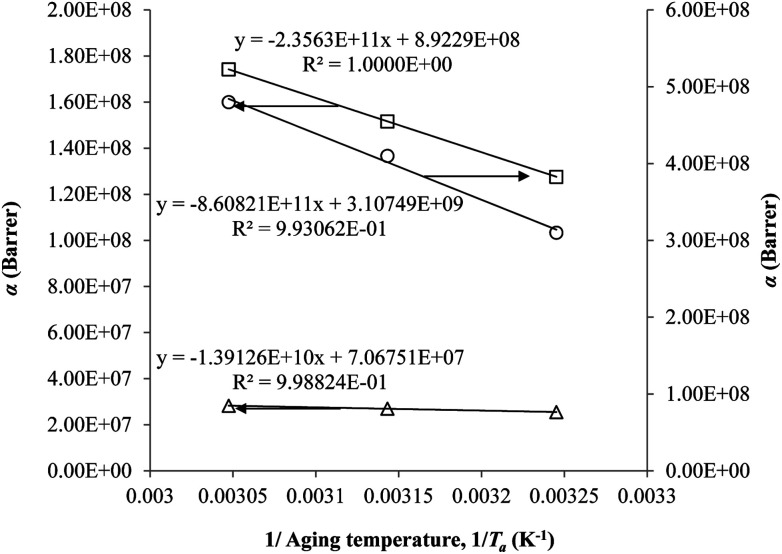
Arrhenius aging temperature, *T*_a_, plots for *α* of oxygen (○) and nitrogen (□) gas permeability data in PSF membrane and oxygen (△) in PPO membrane.

The parameter, *α*, is depicted to similarly exhibit Arrhenius dependency towards aging temperature, such as that shown in Fig. 10, since permeability is a function of temperature and thereby obeys Arrhenius equation.^65–67^ Values of *α* is higher for PSF since it has been reported to be more sensitive to alteration in aging temperature in comparison to PPO.^62^

On the other hand, the *β* parameter has been found to be independent of aging temperature, since it has been reported to be merely related to gas penetrants kinetic diameter.^68–70^ Accordingly, magnitude of the parameter is in accordance with molecular size of the gas molecules, whereby N_2_ (3.64 Å) is larger than O_2_ (3.46 Å).^36^ For, PPO, the value of *β* is larger than its counterpart PSF for a particular gas penetrant since PPO is more dependent on the required volume of void space within the polymeric membrane attributed to its high free volume nature.

**Fig. 11:**
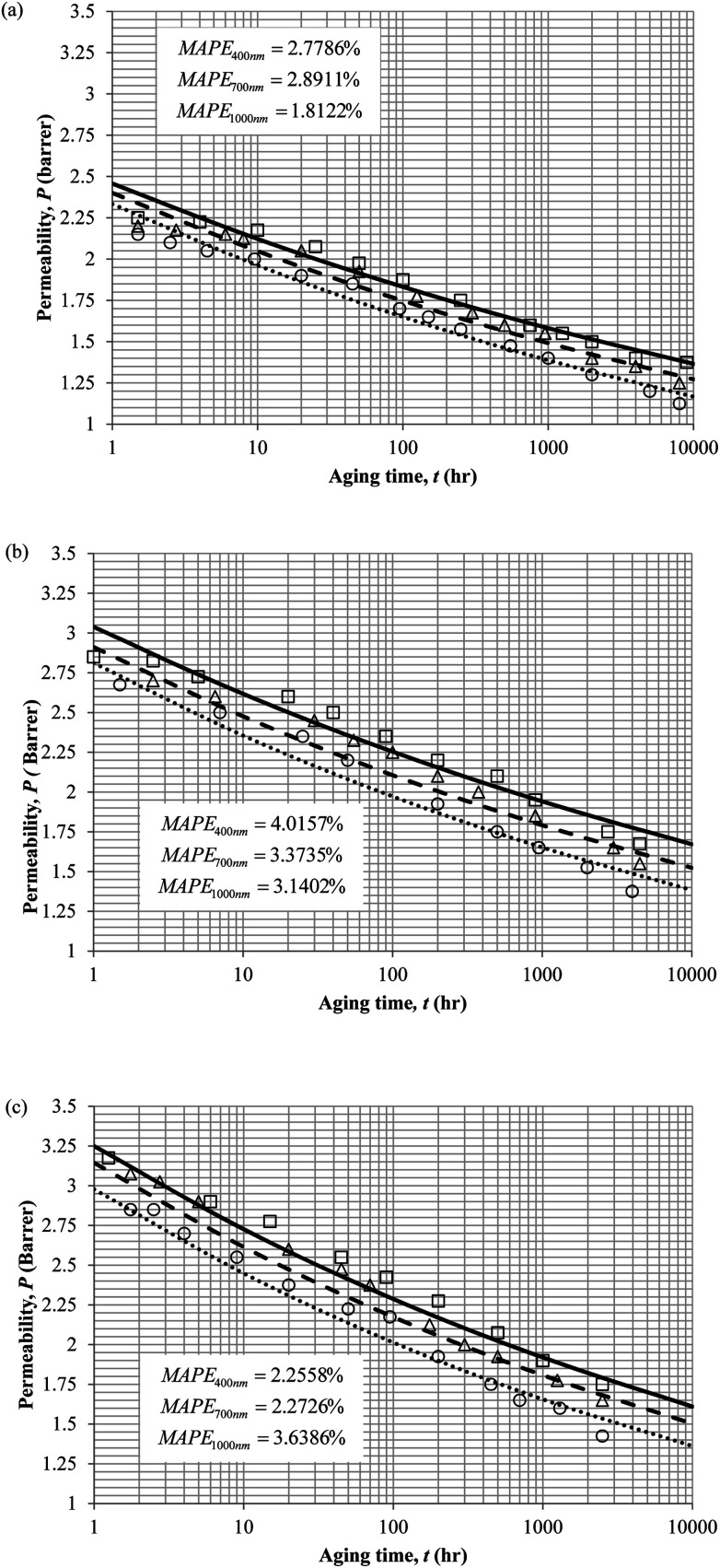
Model validation with oxygen gas permeability data, *P*, by Huang *et al.* (2005) of PSF membrane film during aging time, *t*, with thicknesses of ∼400 nm (○), ∼700 nm (△) and ∼1000 nm (□) with simulation results of ∼400 nm (⋯), ∼700 nm (- - -) and ∼1000 nm (—) at temperature (a) 35 °C (b) 45 °C and (c) 55 °C.

**Fig. 12:**
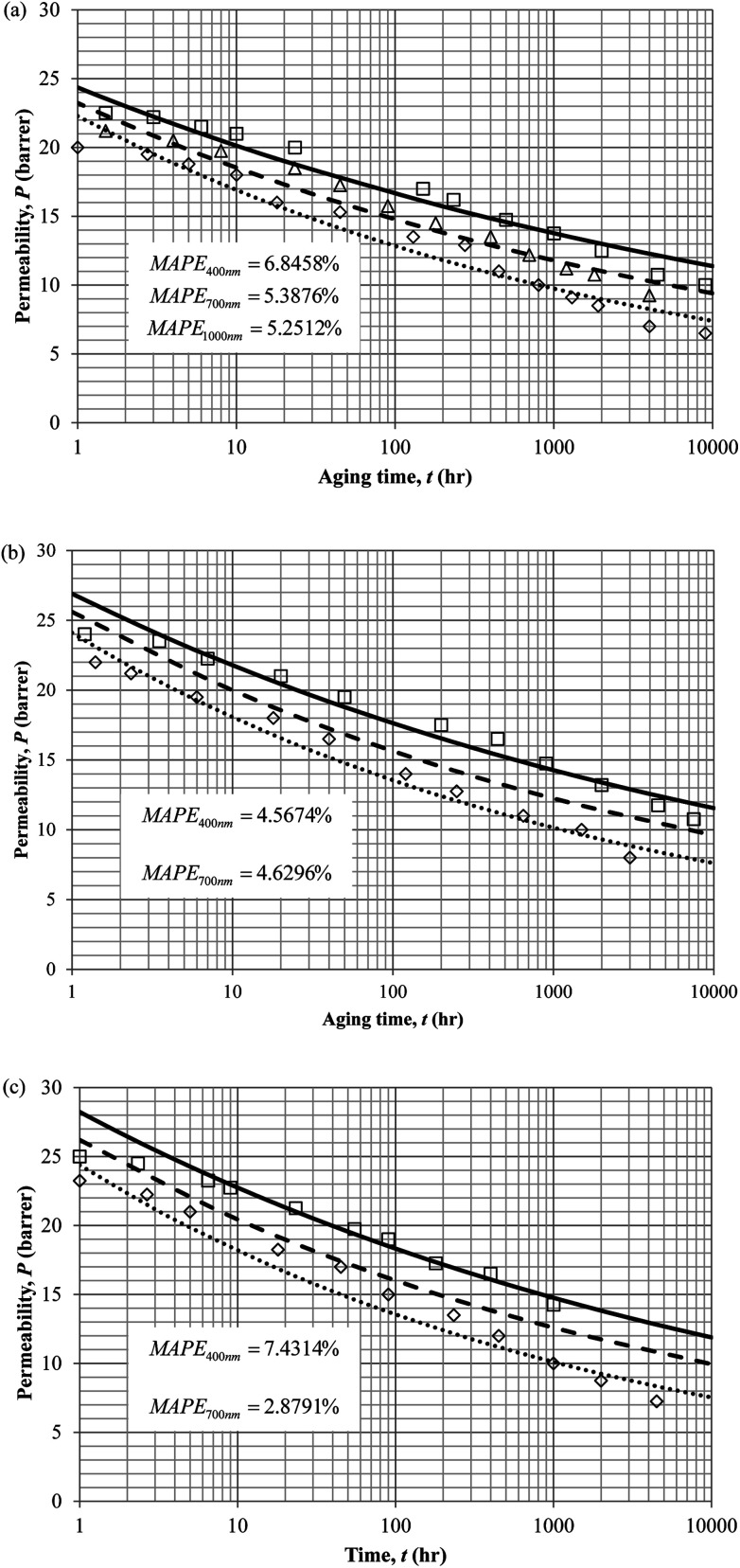
Model validation with oxygen gas permeability data, *P*, by Huang *et al.* (2005) of PPO membrane film during aging time, *t*, with thicknesses of ∼400 nm (○), ∼700 nm (△) and ∼1000 nm (□) with simulation results of ∼400 nm (⋯), ∼700 nm (- - -) and ∼1000 nm (—) at temperature (a) 35 °C (b) 45 °C and (c) 55 °C.

The model validation results for aged gas permeability data by Huang *et al.* (2005)^22,48^ have been depicted Fig. 11(a), (b) and (c) for temperature of 35 °C, 45 °C and 55 °C to represent the transport property of oxygen in PSF polymeric membrane. On the other hand, the model validation of gas permeability of oxygen in PPO membrane at 35 °C, 45 °C and 55 °C has been provided in Fig. 12.

**Fig. 13:**
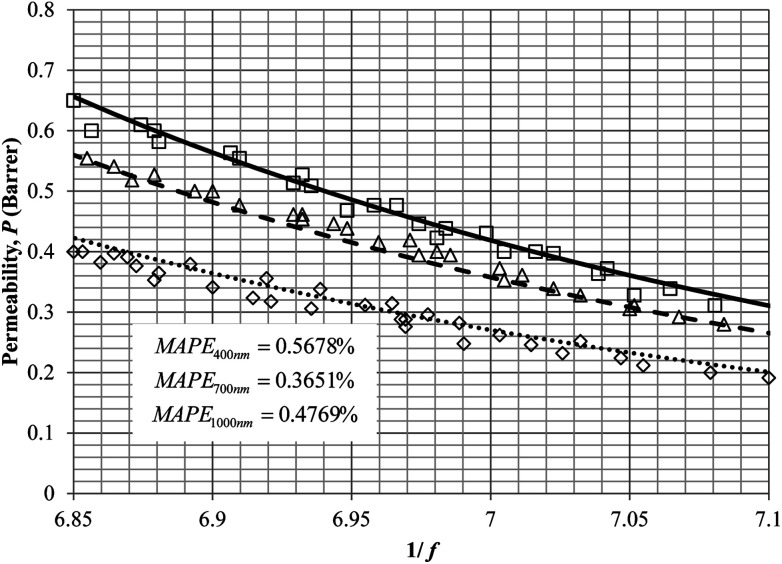
Model validation with nitrogen gas permeability data, *P*, by Huang *et al.* (2005) of polysulfone membrane film with respect to reciprocal of fractional free volume, 1/*f* for thickness of ∼1000 nm at operating temperature of 35 °C (○), 45 °C (△) and 55 °C (□) with simulation results of 35 °C (⋯), 45 °C (- - -) and 55 °C (—).

**Fig. 14:**
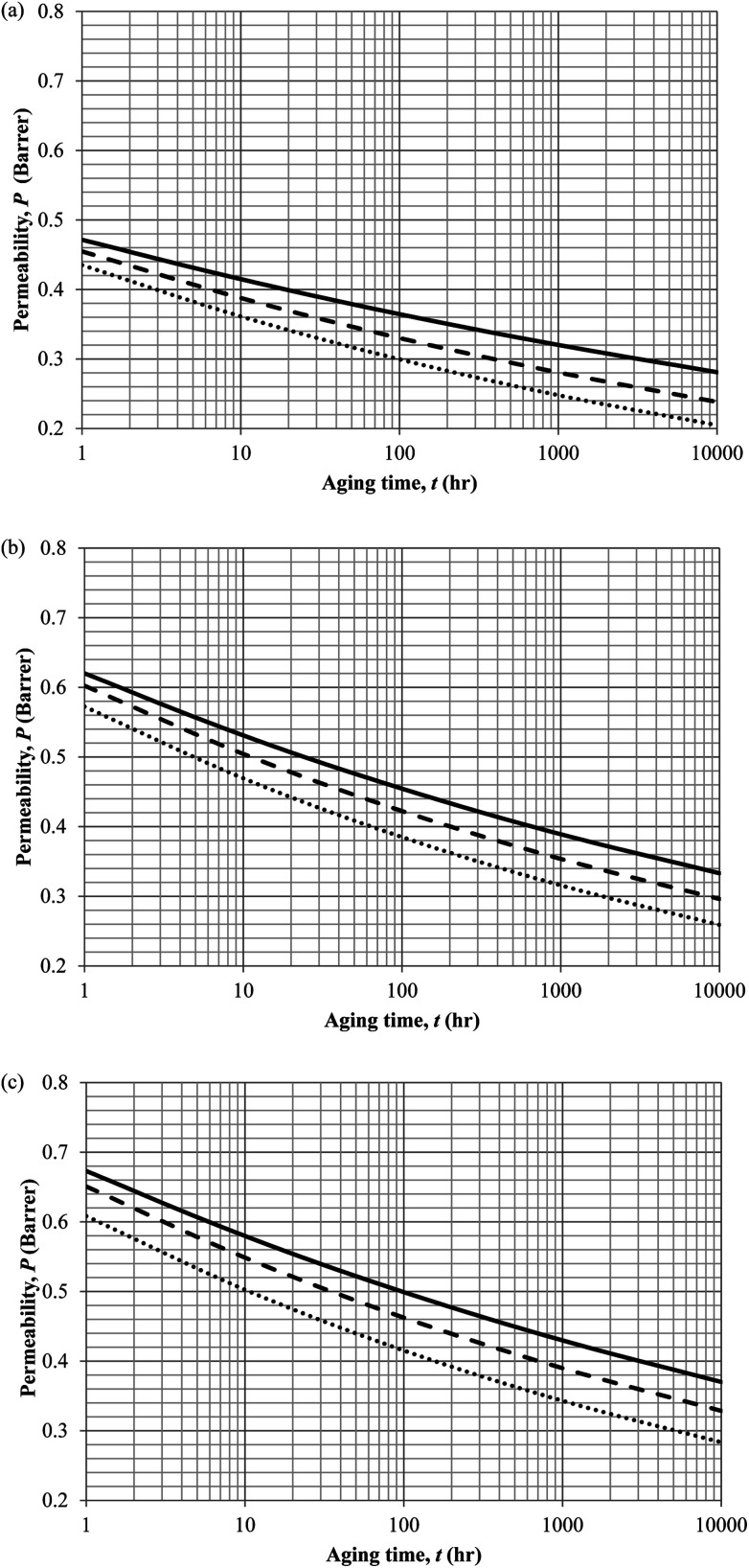
Effect of aging time, *t*, towards the gas permeability, *P*, simulation results of ∼400 nm (⋯), ∼700 nm (- - -) and ∼1000 nm (—) at temperature (a) 35 °C (b) 45 °C and (c) 55 °C.

Similarly, the model validation for gas nitrogen with respect to reciprocal of fractional free volume at different operating temperatures has been provided in Fig. 13, while the effect of aging time to gas permeability of nitrogen at varying membrane thicknesses and operating temperatures based on the parameters in Table 4 has been depicted in Fig. 14.

It is found from Fig. 11 and 12 (gas permeation of O_2_ in PSF and PPO respectively) and Fig. 13 (gas permeation of N_2_ in PSF) that the mathematical model is able to produce good consistency with observation of the physical aging phenomenon for membrane gas transport property in actual laboratory scale. A small MAPE of <5% for PSF membrane and <8% for PPO membrane has been observed over a wide range of film thicknesses at different operating temperature, which reconfirms accuracy of the developed mathematical model. The small discrepancy might be due to experimental error or precision limitations of instruments to measure the amount of permeate across the membrane. Consequently, based upon the physical parameters obtained *via* curve fitting in Fig. 13, the effect of aging time towards nitrogen gas permeability at varying film thicknesses and operating temperature has been predicted and tabulated in Fig. 14.

Permeability of the gases is found to be decreasing with aging time at all temperatures attributed to polymer densification accompanying physical aging that reduces free volume within the polymeric matrix, which in turn confines the void space that serves as passage for diffusivity and solubility of gas penetrants. In addition, permeability of the gas molecules through PSF membrane has been demonstrated to exhibit thickness dependency, with the gas permeability being consistently lower with decrement in film thickness ascribed to enhanced mobility of free volume in the vicinity of a free surface. Other than that, the permeability at a particular aging time in polymeric membranes exposed to higher operating temperature is higher attributed to increment in free volume within the structure of the polymer. Once the free volume of a polymer increases, it allows higher diffusivity of bigger molecules to have a bigger energy to pass through.^71^ All these phenomena, which are consistent to the trend observed in actual experimental condition, have been quantified adequately through the mathematical model.

**Fig. 15:**
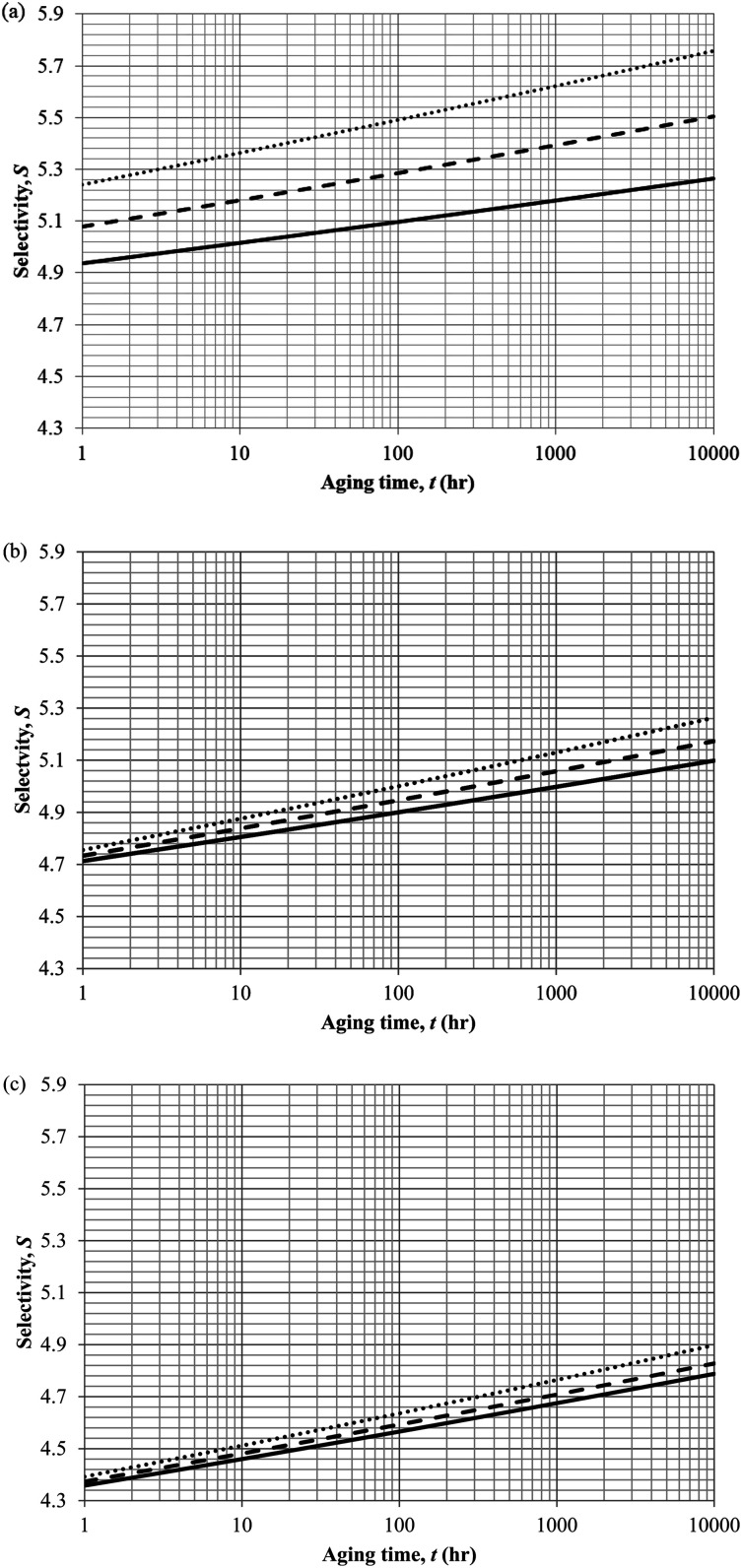
Effect of aging time, *t*, towards the gas selectivity of O_2_/N_2_, *S*, simulation results of ∼400 nm (⋯), ∼700 nm (- - -) and ∼1000 nm (—) at temperature (a) 35 °C (b) 45 °C and (c) 55 °C.

In actual membrane gas separation, other than gas permeability that characterizes the flux of permeation across the membrane barrier, the selectivity is another important parameter since it quantifies the sieving capacity of a membrane by allowing the transport of designated gas molecules, while retaining the movement of its counterpart. Fig. 15 depicts the effect of aging time to the selectivity of PSF polymeric membrane at different thicknesses and operating temperatures.

It is found that the selectivity increases over time attributed to the larger reduction in gas N_2_ as compared to O_2_. Nitrogen demonstrates a higher decrement in gas permeability value with respect to aging time in comparison to oxygen owing to the disparity among their kinetic sizes. The trend is intuitively reasonable since any constriction in molecular spacing within the PSF polymeric membrane matrix is expected to exhibit larger effect on the permeability of bigger gases as compared to its small counterpart. The increment in gas selectivity is more apparent in polymeric membrane of smaller thickness due to the accelerated aging phenomenon in thinner structure that constitute to film structure with smaller cavity sizes and free volume at any particular aging time, further enhancing the sieving capability of the membranes to allow transport of O_2_ with smaller kinetic diameter, while retaining the movement of N_2_. Additionally, the O_2_/N_2_ membrane selectivity is found to be larger for thinner polymeric membrane structure, which can be rationalized through the augmented lost of free volume through enhanced vacancy diffusion mechanism.

With regards to the effect of operating temperature, it is found that the gas selectivity is higher in membrane operated at lower operating temperature, while the enhancement diminishes with increment in operating temperature. The observation can be rationalized through expansion in the cavity sizes that form the channels for transport of gas molecules when operating temperature is raised attributed to higher activation energy for relaxation. The bigger void spaces reduce the sieving capacity of the membrane since it permits gas molecules of different penetrant sizes to transport across the membrane. In addition, the effect of operating temperature to gas selectivity is found to be typically apparent within membrane at lower operating temperature, which can be explained through the fact that the increment in cavity sizes with increment in operating temperature exceeds the kinetic diameter of both O_2_ and N_2_ gases, thus induces minimal effect to the sieving property. Furthermore, the difference among the gas selectivity at different membrane thicknesses is found to be particularly apparent with increment in aging time, which has been attributed to densification throughout the course of aging that enhances permeation of O_2_ but not N_2_.

## 4. Conclusion

In this work, a simple linear correlation has been adapted to provide quantitative explanation and insights towards the aging phenomena at various temperatures and thicknesses through employment of the PVT equation of states to describe the bulk polymer at glass and melt conditions, as well as deviation of thin state from its bulk counterpart. These thermodynamic quantifications have been achieved *via* adaptation of the Tait equation and thickness dependent glass transition temperature respectively. Consequently, the parameters can be employed within a linear correlation alongside the revised WLF equation that quantifies the time to achieve equilibrium to characterize the aging evolution at different operating temperatures. The outcome of this research work is expected to provide a simple characterization of the physical aging process of thin films from the thermodynamic point of view. In addition, the main contribution of the mathematical model is it can successfully isolate the effect of thickness dependent glass transition temperature adopting temperature dependent parameters and thermodynamic relaxation behaviour employing thickness dependent parameters of the modified WLF equations on the latter. The parameters can be quantified through empirical relationship at different film thicknesses and operating temperatures in order to interpolate and predict physical aging performance of different membrane system. Based on findings of present mathematical model, it is found that membrane should be fabricated at lower free volume, greater membrane thickness and operated under condition of lower operating temperature to exhibit the least deteriorating effect in physical aging to ensure minimal fluctuation to membrane separation performance over time. Nonetheless, fabrication and operation of polymers at such features is known to decrease the gas transport property. Lower free volume polymer confines the void spaces of gas penetrants within the membrane matrix, bigger membrane thickness increases the pathway of resistance for permeation while lower operating temperature decreases the activation energy for gas molecules to execute diffusional jump. Realizing that there is a trade-off between polymer membranes with higher gas permeation properties and accelerated aging, it is important to incorporate the developed mathematical model in present work together with conventional models that analytically addresses the separation mechanism of membrane, such as the solution-diffusion equation, typically within industrial process simulator. It is anticipated to be included within process simulation of oxygen enriched combustion using thin polymeric membranes to quantify the impact of physical aging at different operating temperatures and thicknesses towards the gas separation performance and process economics. Consequently, the study can be used to optimize the physical parameters and operating conditions that are most profitable for membrane operation throughout the lifespan of membranes.

## Conflicts of interest

There are no conflicts of interest to declare.

## Nomenclature

*V*(*P*,*T*): Specific volume of the polymer at a particular temperature,*T*, and pressure,*P*
*T*
: Temperature
*P*
: Pressure
*V*(0,*T*): Volume–temperature isotherm at zero pressure
*B*(*T*): Tait parameter
*V*_initial_glass,b_(*p*,*T*_a_): Initial specific volume of bulk polymer state at aging temperature, *T*_a_, and operating pressure,*p*
*T*_a_
: Aging temperature
*p*
: Operating pressure under study
*m*_b_
: Slope of the thermodynamic line characterizing the bulk polymer state
*V*_melt,b_(*p*,*T*_g,b_): Specific volume of bulk polymer melts at bulk glass transition temperature *T*_g,b_ and operating pressure, *p*
*T*_g,b_
: Glass transition temperature of bulk polymer
*T*_g_(*l*): Glass transition temperature of the thin polymeric membrane dependent upon its thickness *l*
*l*
: Thickness of the polymeric film
*ζ*(*T*): Characteristic length of a specific polymer that is temperature dependent
*σ*(*T*): Exponent variable of a specific polymer that is temperature dependent
*m*_actual_
: Slope of the thermodynamic line characterizing actual glassy state
*C*_actual_
: Intercept of the thermodynamic line characterizing actual glassy state
*V*_melt_(*p*,*T*_g_(*l*)): Specific volume of the thickness dependent film at operating pressure, *p*, actual glass transition temperature, *T*_g_(*l*), under melt condition
*V*_initial_glass_(*p*,*T*): Actual initial glassy state for at operating pressure, *p*, and temperature, *T*
*P*(*t*,*T*): Gas permeability, which is time, *t*, and temperature, *T*, dependent
*x*(*T*): Slope in the log–log plot at different operating temperature *T*
*y*(*T*): Constant of the intercept in the log–log plot at different operating temperature T
*f*(*t*,*T*): Fractional free volume, which is time, *t*, and temperature, *T*, dependent
*α*
: Pre-exponential factor in gas permeability-free volume correlation
*β*
: Factor for minimum volume of fluctuation needed for diffusion jump in gas permeability-free volume correlation
*V*(*t*,*T*): Specific volume of the glassy polymeric membrane at a specific time, *t*, and temperature, *T*
*V*_0_
: Specific occupied volume of polymer chain
*V*_w_
: van der Waals volume of the functional group within the polymer repeat unit
*V*_e_(*t*_e_,*T*_a_): Equilibrium specific volume at equilibration time, *t*_e_ and aging temperature, *T*_a_
*t*_e_
: Time to achieve equilibrium
*a*_T_
: Shift factor that describes the relaxation mechanism of the polymer in the WLF equation
*C*_1_, *C*_2_: Constants in the WLF equation

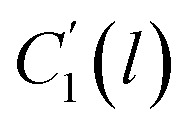
, 
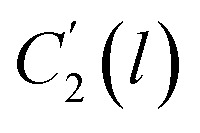
Thickness dependent constants in the revised WLF equation
*m*_aging_
: Slope of the aging line
*t*_0_
: Initial aging time
*C*_aging_
: Constant of the aging line
*x*_sim_
: Simulated data
*x*_exp_
: Experimental data
*N*_exp_
: Number of collected experimental data

## Supplementary Material
